# Single axillary incision endoscopic surgery and liposuction for gynecomastia

**DOI:** 10.1097/MD.0000000000033020

**Published:** 2023-02-17

**Authors:** Mustafa Tukenmez, Selman Emiroglu, Erol Kozanoglu, Bora Edim Akalin, Baran Mollavelioglu, Neslihan Cabioglu, Mahmut Muslumanoglu

**Affiliations:** a Department of General Surgery, Istanbul Faculty of Medicine, Istanbul University, Istanbul, Turkey; b Department of Plastic and Reconstructive Surgery, Istanbul Faculty of Medicine, Istanbul University, Istanbul, Turkey; c Polatli Duatepe State Hospital, Department of General Surgery, Ankara, Turkey.

**Keywords:** endoscopic mastectomy, gynecomastia, liposuction, single incision

## Abstract

Gynecomastia is a common type of breast tissue hypertrophy in men. Surgical excision is the most effective treatment for this condition. Minimally invasive surgical techniques can be used to avoid visible chest scarring. In this study, we evaluated the efficacy and safety of single-axillary-incision endoscopic mastectomy and liposuction for the treatment of gynecomastia. Nipple-sparing mastectomy via a single-port axillary incision was successfully performed in all patients. Twenty-four bilateral procedures were performed in total. Twenty patients underwent liposuction concomitantly. The median weight of the mastectomy pieces was 88.5 g (range: 42.5–440 g), and the median amount of liposuction was 262.5 cc (range: 25–350 cc). The median duration of surgery was 120 minutes (range, 73–195 minutes). Two patients developed a seroma, and 1 patient developed a hematoma in the early postoperative period. The mean satisfaction levels related to physical appearance, mental status, and social environment were 8.75 (standard deviation [SD]: 1.19), 9.17 (SD: 1.44), and 9.33 (SD: 0.76) points, respectively, on a 10-point visual analog scale. Endoscopic single-port nipple-sparing mastectomy combined with liposuction is a technically feasible method to avoid anterior chest wall scarring with good cosmetic results. Between June 2021 and June 2022, 30 patients underwent endoscopic single-port nipple-sparing mastectomy through a small axillary incision, while 20 underwent concomitant liposuction. The demographic information of the patients, duration of surgery, amount of tissue removed, and complications were recorded. Patients’ levels of satisfaction with their physical appearance, mental status, and social environment were measured.

## 1. Introduction

Gynecomastia is a benign enlargement of the male breast due to the proliferation of the breast tissue. Physiologic gynecomastia is common in neonates, adolescents, and elderly men.^[1]^ Even if gynecomastia is diffuse and asymptomatic, careful anamnesis, physical examination, and laboratory analysis should be performed to rule out any underlying breast tumor or endocrine or systemic disease. Gynecomastia often resolves spontaneously, and these cases require careful clinical observation. A small number of patients require treatment for cosmesis and analgesia. The earlier the medication is started, the more effective it becomes. Surgery is useful for patients who have been symptomatic for a long time, and for whom medical treatment has been ineffective.^[1,2]^

Many open surgical techniques are used to treat gynecomastia. Complications such as subtotal glandular resection, contour irregularity, nipple–areola complex collapse, nipple–areola complex distortion, ischemia, necrosis, and diffuse scarring can occur.^[3–5]^ A minimally invasive approach has been used in breast surgery for both malignant and benign diseases such as gynecomastia.^[6–9]^ Minimally invasive techniques for gynecomastia initially include the use of vacuum-assisted biopsy devices and liposuction from the anterior axillary line and inframammary fold.^[10,11]^ Endoscopic mastectomy techniques have been explored over the last decade.^[12–18]^

This study aimed to investigate the efficacy of endoscopic mastectomy and liposuction through a single axillary incision, and its effects on patient satisfaction.

## 2. Patients and Methods

The study was approved by the local ethics committee (Istanbul Medical Faculty Clinical Research Ethics Committee Number: E-29654016-050.99-1105820). Informed consent was obtained from all the patients. Thirty-four patients were scheduled to undergo gynecomastia surgery between June 2021 and June 2022. Detailed anamnesis, physical examination, breast ultrasonography, and the necessary laboratory tests were performed preoperatively. Four patients were excluded from the study because they required skin excision. A total of 54 endoscopic mastectomies were performed, 24 bilaterally and 6 unilaterally. Twenty patients underwent liposuction concomitantly. Patient satisfaction related to physical appearance, mental status, and social environment were evaluated using a visual analog scale (VAS) at the 3-month follow-up.

### 2.1. Surgical technique

Under general anesthesia with endotracheal intubation, the patients were placed in the supine position on an operating table with the arm abducted.

**Figure 1.**
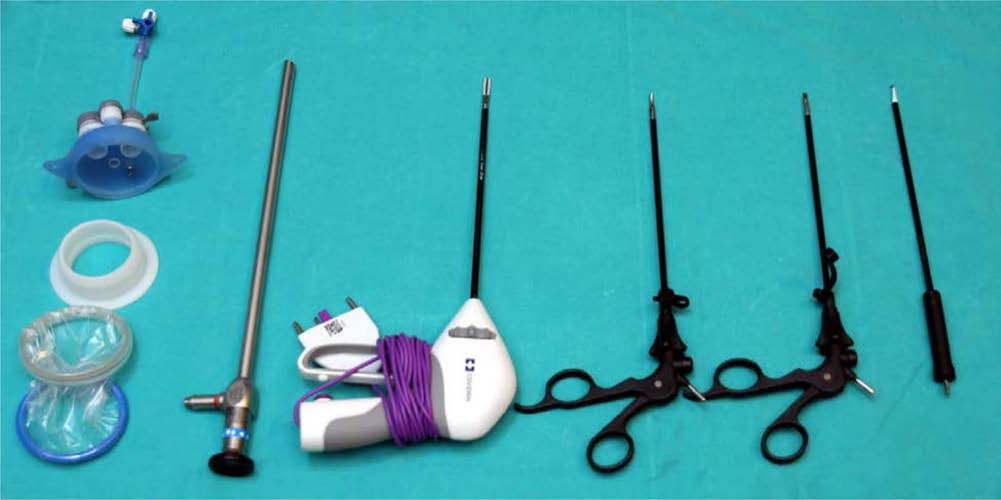
Instruments.

First, a 3-cm long axillary incision was made, and the lateral aspect of the pectoralis major muscle was accessed through this incision. The subcutaneous flap was prepared to be as wide as possible under direct visualization to avoid a blind spot in front of the port. A space is created to insert a single port. A single incision laparoscopic surgery port (equipped with two 10-mm and two 5-mm ports) was introduced into the same incision. An insufflator was connected to the single incision laparoscopic surgery port and set at a pressure of 8 mm Hg. Carbon dioxide was inflated to a pressure of 8 mm Hg. It helped separate the gland from the fat of the subdermal layer and facilitated dissection of the Cooper’s ligament. A single port was introduced with a 10-mm 30-degree camera. Two 5-mm ports were used to insert an Endo Grasp and LigaSure device. The optical system and the entire endoscopic system were obtained from a laparoscopy instrumentation box (Fig. 1).

First, superficial dissection was performed from the axilla to the nipple, and breast and subcutaneous tissues were separated using LigaSure. Subsequently, the area from the nipple to the inframammary fold was dissected both laterally and medially. After the subcutaneous tissue was excised, the breast tissue was dissected over the pectoralis major muscle in the deep plane. The inframammary fold tissue inferior to the breast was also dissected, and the breast tissue was completely mobilized. The breast tissue was removed through the axillary incision and sent to the pathology department with routine directional markings. All patients were cosmetically evaluated preoperatively and peroperatively by a plastic and reconstructive surgery team independent of the mastectomy team. Twenty patients underwent liposuction performed by the plastic and reconstructive surgery team, considering that liposuction would make an additional contribution.

**Figure 2.**
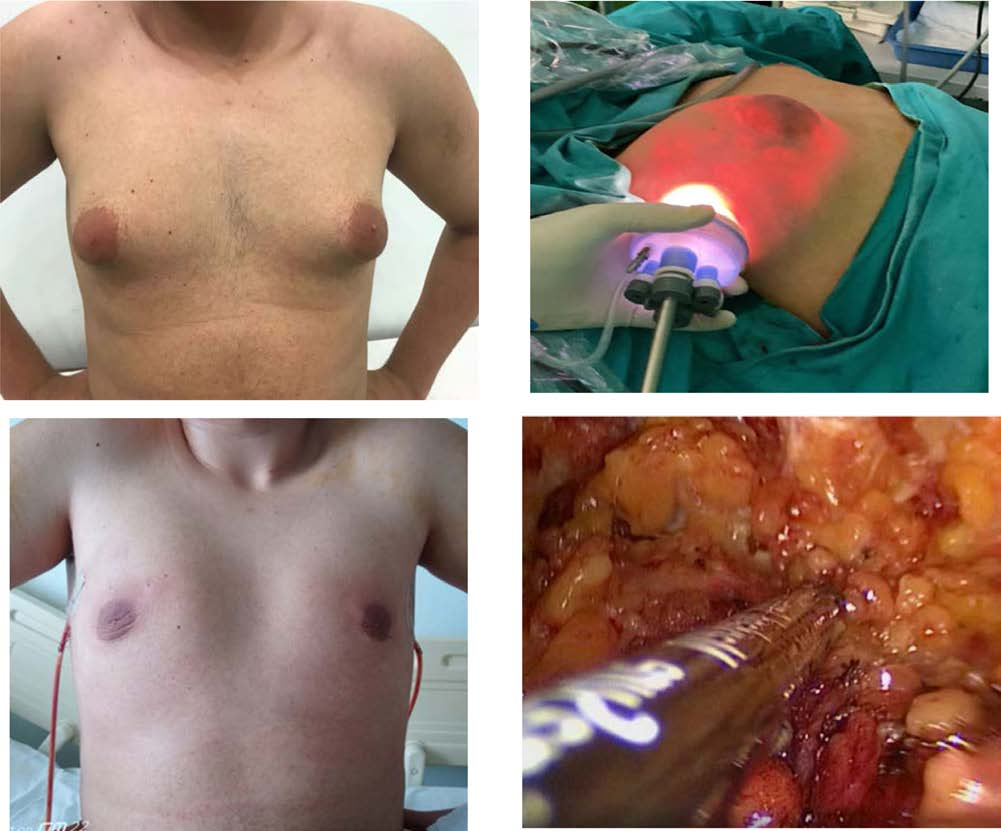
Preoperative, peroperative, and postoperative photographs.

At the end of the mastectomy, an infiltration solution was introduced through the mastectomy incision using an infiltration cannula. The infiltration solution was prepared at a volume of 1000 mL with 0.9% NaCl, to which 400 mg of lidocaine and 1 mL of adrenaline were added. Each breast was infiltrated with 250 to 350 mL of the infiltration solution according to wet liposuction principles, and a 10-minute waiting time was maintained before the procedure. The liposuction cannula was introduced through the mastectomy incision, and conventional non-power-assisted liposuction was performed for each breast. The mastectomy flaps were compressed against the ipsilateral pectoralis major muscles to maintain negative pressure of liposuction. Caution was taken to prevent any type of depression under both the nipple–areola complex and the mastectomy flaps. At the end of the procedure, each breast was palpated for asymmetries and subcutaneous roughness, and if present, such deformities were corrected with additional liposuction. The operation ended with the placement of 1 suction drain per breast, following which wound closure was performed after resection of 1-mm incision margins (Fig. 2). Compression dressings were placed over the breasts to obliterate the dead space, and the patients were advised to wear them for 3 months. The dressings were worn continuously for the first 6 weeks, after which they began to wear the dressings during daily activities for the next 6 weeks.

### 2.2. Statistical analysis

Statistical Package for the Social Sciences version 25.0 (IBM Corp., Armonk, NY) was used for statistical analysis. The conformity of continuous variables to a normal distribution was analyzed using descriptive, graphical, and statistical methods. The Shapiro–Wilk test was used to test the normality of the scores obtained from a continuous variable using statistical methods. Patient satisfaction levels related to physical appearance, mental status, and social environment were evaluated using the 10-point VAS. In addition to descriptive statistical methods (number, percentage, mean, median, standard deviation, etc.), the relationships between quantitative variables were analyzed using the Spearman correlation test. The Mann–Whitney *U* test was used to compare quantitative variables between the 2 groups. The Pearson chi-square test was used for qualitative comparisons between groups. Results were evaluated at a 95% confidence interval, with a *P* value of < .05, considered statistically significant.

## 3. Results

### 3.1. Patient characteristics

A total of 30 male patients were included in the study. Their median age was 31 years (range: 18–53), and their median body mass index (BMI) was 26.2 (range: 23.7–37.9). Of the 30 patients, 53% were overweight and 20% were obese. Bilateral endoscopic mastectomy (EM) was performed in 24 patients (80%), unilateral EM in 6 patients (20%), and liposuction with EM in 66.7% (n = 20). The median weight of the mastectomy piece removed during the surgical procedure was 88.5 g (range: 42.5–440 g) and the median amount of liposuction was 262.5 cc (range: 25–350 cc). The median operative time was 120 minutes (range: 73–195 minutes). In the early postoperative period, minor complications that did not require hospitalization occurred in 3 patients (10%), including seroma in 2 (6.7%) patients and hematoma in 1 (3.3%) patient (Table 1).

**Table 1:** Patient characteristics (n = 30).

Characteristics	n (%)	Median (minimum–maximum)
Age	30 (100)	31 (18–53)
BMI	30 (100)	26.2 (23.7–37.9)
BMI group		
Normal	8 (26.7)	
Overweight	16 (53.3)	
Obese	6 (20)	
Breast surgery		
Unilateral	6 (20)	
Bilateral	24 (80)	
Total amount of breast tissue removed (g)	30 (100)	88.5 (42.5–440)
Liposuction amount (cc)	20 (66.7)	262.5 (25–350)
Duration of surgery (min)	30 (100)	120 (73–195)
Complication		
Hematoma	1 (3.3)	
Seroma	2 (6.7)	
No complications	27 (90)	

BMI = body mass index.

### 3.2. Patient satisfaction (VAS) scores

The mean satisfaction levels related to physical appearance, mental status, and social environment were 8.75 (standard deviation [SD]: 1.19), 9.17 (SD: 1.44), and 9.33 (SD: 0.76) points, respectively, on a 10-point VAS (Table 2).

**Table 2:** Patient satisfaction (VAS) scores.

Satisfaction	Mean (SD)	Minimum–maximum
Physical appearance	8.75 (1.19)	7–10
Mental state	9.17 (1.44)	5–10
Social environment	9.33 (0.76)	8–10

SD = standard deviation, VAS = visual analog scale.

### 3.3. Relationship between continuous variables and comparison of categorical data

A significant positive correlation was observed between age and satisfaction with physical appearance (*R* = 0.521; *P* = .009), BMI and satisfaction with social environment (*R* = 0.489; *P* = .015), and weight of breast tissue removed during surgery and mental satisfaction (*R* = 0.425; *P* = .039). Based on these findings, it was determined that satisfaction with physical appearance increased with increasing age, satisfaction related to social environment increased with increasing BMI, and satisfaction related to mental status increased with the increasing amounts of breast tissue removed perioperatively. The relationships between the other variables are presented in detail in Table 3. There was no significant difference in the age, BMI, and postoperative satisfaction level of the patients with respect to the presence or absence of concomitant liposuction (*P* > .05; Table 4).

**Table 3:** Relationship between continuous variables.

No.	Variables	No.						
		1	2	3	4	5	6	7
1	Age	NA						
2	BMI	0.041						
3	Total amount of breast tissue removed (g)	0.204	0.546*					
4	Liposuction amount (cc)	−0.040	0.646*	0.201				
5	Physical appearance	0.521*	−0.159	−0.019	0.096			
6	Mental state	0.303	0.031	0.425†	−0.289	0.596*		
7	Social environment	0.149	0.489†	0.206	0.354	0.453†	0.687*	NA

NA = not available.

* *P* < .01, Spearman correlation analysis.

† *P* < .05, Spearman correlation analysis.

**Table 4:** Patient data with respect to the presence of absence of liposuction.

Variables	Liposuction		*P* value
	Yes (n = 20)	No (n = 10)	
Age, mean (SD)	31.90 (10.02)	34.20 (14.26)	.860*
BMI, mean (SD)	27.76 (4.07)	26.82 (2.61)	.597*
BMI group, n (%)			
Normal	4 (20)	4 (40)	.472†
Overweight	12 (60)	4 (40)	
Obese	4 (20)	2 (20)	
Physical appearance, mean (SD)	8.50 (1.03)	9.25 (1.39)	.153*
Mental state, mean (SD)	9.38 (0.72)	8.75 (2.32)	.581*
Social environment, mean (SD)	9.38 (0.72)	9.25 (0.89)	.789*

*P* > .05.

BMI = body mass index, SD = standard deviation.

* Mann–Whitney *U* test.

† Chi-square test.

## 4. Discussion

Although EM and endoscopic breast surgery were described in the 1990s,^[19,20]^ these procedures did not achieve widespread clinical use because neither the technique nor the devices were sufficiently effective. EM has evolved in recent years. A wide range of instruments ranging from long facelift scissors to various types of retractors has been used under direct visualization with an endoscope.^[9]^ Owing to the introduction of single-port and vessel-sealing devices, axillary EM has been used by surgeons experienced in endoscopic surgery.^[21]^

Today, many surgical techniques are used for gynecomastia, such as periareolar, transareolar, and inframammary incisions and skin excision techniques. Breast amputation using the free nipple graft technique is used in patients who require excessive skin excision.^[4]^ Ohyama et al^[20]^ performed endoscope-assisted transaxillary glandular tissue removal in gynecomastia.

Jarar et al^[6]^ made a 15-mm incision from the anterior axillary line at the nipple level, injected lipolysis solution, and then performed liposuction. Through the same incision, the remaining fibroglandular tissue was excised by entering with an endoscope while the surgical assistant manually elevating the skin. Yang et al^[14]^ performed endoscopic subcutaneous mastectomy in 45 gynecomastia cases. They performed liposuction by first injecting tumescent solution through a 3-cm axillary incision, then creating a suitable surgical site with a retractor, and then performing sharp dissection using Metzenbaum scissors and other instruments. In our study, dissection could be performed from the correct plane owing to the easy visualization of Cooper’s ligament with the help of CO_2_ insufflation with a single port without using any retractors. In our technique, flaps were formed by creating a pneumocopper with a CO_2_ pressure of 8 mm Hg. LigaSure was used for dissection to minimize bleeding. Fibroglandular tissue was excised first, and then if there was excess fat tissue around the breast tissue, a tumescent solution was administered and liposuction was performed. Using our combined technique, favorable cosmetic results can be achieved.

Varlet et al^[13]^ performed endoscopic subcutaneous mastectomy with one 10-mm port and two 5-mm ports in 12 patients with adolescent gynecomastia and removed the breast tissue in small pieces, which prolonged the operation time (93.3 minutes for each side). In our technique, the operation time was shorter as there was no need for extra time for breast tissue removal because of the use of a single port.

An important technical point to be considered is that dissection should be started and continued from the superficial plane first, and then switched to the deep plane. Otherwise, superficial dissection may become difficult owing to the excessive mobilization of the fibroglandular tissue.^[9]^

Liposuction is used alone or in combination with other techniques in gynecomastia surgery.^[4]^ Particularly in cases with excess subcutaneous adipose tissue, after the excision of breast tissue, depression may occur around the nipple. Liposuction can prevent this appearance, correct the asymmetry, and remove the roughness. In this study, although the amount of liposuction increased as BMI increased (Table 3), liposuction had no significant effect on postoperative satisfaction level (Table 4). Performing liposuction according to the preoperative and perioperative evaluation of cosmetic results positively affected patient satisfaction in terms of physical appearance, mental status, and social environment.

Patients with gynecomastia experience anxiety, depression, social phobia, and psychological anguish. A successful surgery has a positive effect on psychological well-being as well as cosmetic correction of the chest area.^[22]^ In this study, mental and social satisfaction levels were high in patients who underwent gynecomastia surgery. It was found that mental satisfaction increased as the amount of tissue removed increased and social satisfaction increased as patients BMI increased.

Although EM has many advantages over open surgery, such as small incisions, minimal tissue trauma, increased nipple viability, and early recovery, it also has limitations, such as a small surgical site, the use of rigid instruments, instrument collisions, and a prolonged operative time. The limitation of this technique is that it cannot be used in cases that require skin excision. In the near future, the use of angulated flexible instruments closer to a single port and mobile 3-dimensional cameras will reduce instrument collisions and operative time.

## 5. Conclusions

Endoscopic nipple–areola-sparing mastectomy combined with liposuction with a single axillary incision is a safe and effective technique for the surgical treatment of gynecomastia, with high patient satisfaction in terms of physical appearance, mental status, and social environment. Although the operative time is long and the learning curve is steep, it can easily be performed by experienced surgeons as an alternative method for patients who do not require skin excision

## Acknowledgments

The authors are grateful to all colleagues who helped with the preparation of this article.

## Author contributions

**Conceptualization:** Mustafa Tukenmez.

**Data curation:** Mustafa Tukenmez, Selman Emiroglu, Erol Kozanoglu, Bora Edim Akalin, Baran Mollavelioglu.

**Formal analysis:** Mustafa Tukenmez.

**Methodology:** Mustafa Tukenmez.

**Project administration:** Mustafa Tukenmez.

**Supervision:** Mustafa Tukenmez, Neslihan Cabioglu, Mahmut Muslumanoglu.

**Writing – original draft:** Mustafa Tukenmez.

**Writing – review & editing:** Mustafa Tukenmez.
